# *In Vivo* Functional Requirement of the Mouse *Ifitm1* Gene for Germ Cell Development, Interferon Mediated Immune Response and Somitogenesis

**DOI:** 10.1371/journal.pone.0044609

**Published:** 2012-10-24

**Authors:** Ingeborg Klymiuk, Lukas Kenner, Thure Adler, Dirk H. Busch, Auke Boersma, Martin Irmler, Valérie Gailus-Durner, Helmut Fuchs, Nicole Leitner, Mathias Müller, Ralf Kühn, Michaela Schlederer, Irina Treise, Martin Hrabě de Angelis, Johannes Beckers

**Affiliations:** 1 Institute of Experimental Genetics and German Mouse Clinic, Helmholtz Zentrum München GmbH, Neuherberg, Germany; 2 Ludwig Boltzmann Institute for Cancer Research and Institute for Clinical Pathology, Medical University Vienna, Vienna, Austria; 3 Institute for Medical Microbiology, Immunology and Hygiene, Technische Universität München, Munich, Germany; 4 Institute of Laboratory Animal Science and Biomodels Austria, University of Veterinary Medicine Vienna, Vienna, Austria; 5 Institute of Animal Breeding and Genetics, University of Veterinary Medicine Vienna, Vienna, Austria; 6 Institute of Developmental Genetics, Helmholtz Zentrum München GmbH, Neuherberg, Germany; 7 Ludwig Boltzmann Institute for Cancer Research, Ludwig Boltzmann Gesellschaft, Vienna, Austria; 8 Experimental Genetics, Technische Universität München, Freising-Weihenstephan, Germany; Ohio State University Comprehensive Cancer Center, United States of America

## Abstract

The mammalian *Interferon induced transmembrane protein 1 (Ifitm1*) gene was originally identified as a member of a gene family highly inducible by type I and type II interferons. Based on expression analyses, it was suggested to be required for normal primordial germ cell migration. The knockdown of *Ifitm1* in mouse embryos provided evidence for a role in somitogenesis. We generated the first targeted knockin allele of the *Ifitm1* gene to systematically reassess all inferred functions. Sperm motility and the fertility of male and female mutant mice are as in wild type littermates. Embryonic somites and the adult vertebral column appear normal in homozygous *Ifitm1* knockout mice, demonstrating that *Ifitm1* is not essential for normal segmentation of the paraxial mesoderm. Proportions of leucocyte subsets, including granulocytes, monocytes, B-cells, T-cells, NK-cells, and NKT-cells, are unchanged in mutant mice. Based on a normal immune response to *Listeria monocytogenes* infection, there is no evidence for a dysfunction in downstream IFNγ signaling in *Ifitm1* mutant mice. Expression from the *Ifitm1* locus from E8.5 to E14.5 is highly dynamic. In contrast, in adult mice, *Ifitm1* expression is highly restricted and strong in the bronchial epithelium. Intriguingly, IFITM1 is highly overexpressed in tumor epithelia cells of human squamous cell carcinomas and in adenocarcinomas of NSCLC patients. These analyses underline the general importance of targeted *in vivo* studies for the functional annotation of the mammalian genome. The first comprehensive description of the *Ifitm1* expression pattern provides a rational basis for the further examination of *Ifitm1* gene functions. Based on our data, the fact that IFITM1 can function as a negative regulator of cell proliferation, and because the gene maps to chromosome band 11p15.5, previously associated with NSCLC, it is likely that IFITM1 in man has a key role in tumor formation.

## Introduction

The *Interferon induced transmembrane protein 1* (*Ifitm1, fragilis2, mil-2, 9–27, Leu-13*) gene belongs to a family of at least five sequence related genes (*Ifitm1, Ifitm2, Ifitm3, Ifitm5, Ifitm6*) located in a 68 kb gene cluster on mouse chromosome 7. Highly conserved genes are found in man (chromosome 11), rat (chromosome 1), cow (chromosome 29) and other species [1]–[3]. All five mouse *Ifitm* genes are transcribed each from two or three exons and encode small proteins of 5–17 kDa. *Ifitm1* encodes the longest transcript of the *Ifitm* genes and is translated into a 17 kDa protein [4]. All *Ifitm* proteins consist of a short extracellular domain, highly conserved transmembrane and cytoplasmic domains followed by a second transmembrane or membrane-associated domain [5].

Several putative functions have been suggested for the *Ifitm* genes based either on indirect experimental evidence or inferred from sequence or gene expression data. *Ifitm1* was originally identified as an interferon (IFN) induced protein in neuroblastoma cells [6] and the promoters of *Ifitm1, Ifitm3* and *Ifitm6* contain interferon stimulated response elements (ISREs), suggesting that they might be regulated during an antitumoral, antiviral or antibacterial immune response [2], [7]. Several comparative gene expression analyses, mainly in human tissues, revealed alterations of *Ifitm1* expression in various cancer types, including breast cancer, colorectal tumors, gastric cancer, esophageal cancer, ovarian carcinoma, head and neck cancer, pancreatic cancer and lung cancer [8]–[21], as well as in a form of schizophrenia and in Epstein-Barr virus related diseases [13]–[15], [18], [22], [23]. In addition, *Ifitm1* was described as a marker for the prognosis of chronic myeloid leukemia [24]. These observations have led to the suggestion that *Ifitm1* might be expressed as a general response to IFN signaling under various disease conditions [25]–[27].

Another suggested function for *Ifitm1* is its requirement for primordial germ cell (PGC) specification and migration. Expression of *Ifitm1, Ifitm2* and *Ifitm3* was detected in the region of the mouse embryonic epiblast where cells acquire germ cell competence starting from day 7.25 of embryonic development (E7.25). Therefore, *Ifitm1* together with other genes of the family were suggested to be required for PGC development and migration to the genital ridges in mammals [2], [28]–[33]. However, deletion of the entire *Ifitm* gene cluster on mouse chromosome 7 questioned this assumption, since mice with a homozygous *Ifitm* cluster deletion were viable and fertile and underwent normal PGC development [1].

Furthermore, it was suggested that the *Ifitm1* gene might be a downstream target of the *Wnt/β-catenin* signaling pathway, as *β-catenin* activation induced expression of the *Ifitm* genes in mice and human colon carcinoma cells [10]. This was further supported by the finding that *Wnt* signaling influences the induction and migration of PGCs, and that genes of the *Wnt* pathway are co-expressed in some tissues with *Ifitm1* [34]. In a *Wnt/β-catenin* knockout study, *Ifitm1* was also identified as a potential target gene of this cell-signaling pathway [35]. Additionally, a knockdown of *Ifitm1* by RNA interference (RNAi) was characterized by an embryonic phenotype with a kinked neural tube and defects in somite formation at E8.5 [35].

To reconcile some of these conflicting results *in vivo* and to study the function specifically of *Ifitm1* we used a targeted mutagenesis approach that replaced the coding region of the *Ifitm1* gene with the *lacZ* reporter gene in the mouse. We describe the phenotype of this *Ifitm1* loss-of-function allele (*Ifitm1^tm1IEG^*). In particular, we reassess in detail the postulated functions for *Ifitm1* and present for the first time its complex expression pattern during embryogenesis and the restricted expression of *Ifitm1* in adult organs, enabling the analysis of new *Ifitm1* functions. Intriguingly, we find that *Ifitm1*, respectively, the human protein IFITM1 are selectively expressed in the bronchial epithelia of mouse and human lung tissue and that IFITM1 is highly overexpressed in human non-small cell lung cancers (NSCLC).

## Results

### Targeting of the *Ifitm1* Gene in Mouse ES Cells

**Figure 1:**
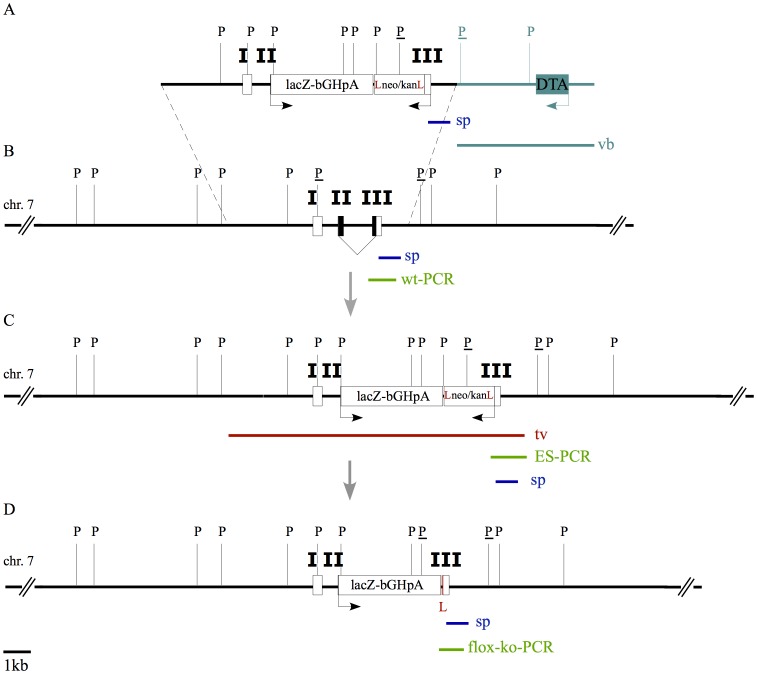
Schematic view of the *Ifitm1* targeting strategy by homologous recombination in mouse ES cells. (**A**) Shows the scheme of the linearized targeting vector used for electroporation in mouse ES cells. The coding sequence of the *lacZ* reporter gene (lacZ) was inserted in frame into the first codon of the *Ifitm1* gene. The three *Ifitm1* exons are indicated as I, II, and III. The *lacZ* gene is followed by a poly-adenylation signal (bGHpA) and a neomycin/kanamycin selection cassette (neo/kan) that was flanked by *loxP* sites (L) for subsequent deletion of the cassette. The backbone of the vector (vb) included the coding sequence for the diphtheria toxin fragment A (DTA) to support the selection of clones carrying the homologous recombination of the targeting vector. The location of the probe used for Southern blotting (sp) and PvuII restriction sites (P) are also indicated. The PvuII restriction sites that are relevant for the diagnostic restriction fragments in Southern blots in combination with DNA probe sp are marked with an underline (P). (**B**) Shows a scheme of the wildtype *Ifitm1* locus on mouse chromosome 7. The three *Ifitm1* exons (I, II, and III) are indicated as boxes. Black shading in the boxes indicates the coding sequence of *Ifitm1*. The genomic region that was amplified by PCR to genotype the *Ifitm1^wt^* allele is indicated (wt-PCR). (**C**) Shows the *Ifitm1* locus following homologous recombination in mouse ES cells. As result of recombination, the complete *Ifitm1* coding sequence is replaced by the *lacZ* reporter gene and the selection cassette (lacZ-bGHpA-Lneo/kanL). The genomic region that is covered by the homologous sequence of the targeting vector (tv) is designated. The genomic region that was amplified by PCR to genotype the targeted *Ifitm* locus in the electroporated ES cells following G418 selection is indicated (ES-PCR). (**D**) Shows a scheme of the *Ifitm1^tm1IEG^* loss-of-function allele that was generated by expression of the *Cre* recombinase and subsequent deletion of the neo/kan selection cassette from the targeted *Ifitm1* allele shown in (**C**). As result of this excision, a single *loxP* site (L) is left behind as indicated. The genomic region that was amplified by PCR to genotype the targeted *Ifitm^tm1IEG^* loss-of-function allele in mice obtained from matings between chimeric mice and *Cre* expressing mice is indicated (flox-ko-PCR). The size bar (bottom left) indicates 1 kb length.

We generated a novel *Ifitm1* knockin mouse line by replacing the coding region of the mouse *Ifitm1* gene with a *lacZ* reporter gene (Fig. 1). After gene targeting with linearized vector DNA we picked 624 G418 resistant ES cell clones. Genomic DNA from these targeted ES cells was used for PCR screening (Fig. S1, panel A), which identified twelve targeted ES cell clones. For three of the 12 clones the correct homologous recombination was subsequently confirmed by Southern blot hybridization using an internal probe (Fig. 1). However, in two clones we identified additional non-homologous integration sites of the targeting vector. Homologously targeted ES cells of the clone without additional transgenic integration site were used for blastocyst injection and production of chimeric mice. Chimeras were then bred with C57BL/6J^Cre/+^ female mice to remove the neomycin selection cassette yielding the *Ifitm1* loss-of-function allele named *Ifitm1^tm1IEG^*. The offspring of these breedings were backcrossed to the C57BL/6J strain for at least five generations and again screened by Southern blot hybridization (Fig. S1, panel B) confirming the proper targeting of the *Ifitm1* gene and the deletion of the neomycin selection cassette.

### Distribution of Genotypes: Mendelian Ratios

We analyzed the distribution of genotypes of the pups of two independent rounds of heterozygous matings. The first set of 11 litters resulted in a total number of 96 pups with 20 homozygous mutant (20.83%), 42 heterozygous mutant (43.75%) and 34 wildtype (35.42%) animals. The second set of 19 heterozygous breeding pairs resulted in a total number of 146 pups with 35 homozygous mutant (23.97%), 84 heterozygous mutant (57.53%) and 27 wildtype (18.49%) animals. Therefore, no evidence for embryonic lethality was found in *Ifitm1^tm1IEG/tm1IEG^* animals.

### Analysis of Sperm Motility and Fertility of *Ifitm1^tm1IEG/tm1IEG^* Animals

Since *Ifitm1* was previously associated with PGC migration and sperm development [2], [28], [31]–[33], we analyzed sperm quality using a comprehensive sperm motility assay. We did not find statistically significant differences in any sperm motility associated parameter between sperm samples from the homozygous *Ifitm1* knockout males (n  = 10) and the wildtype control males (n  = 10) (Fig. S2).

To exclude the possibility that either subtle changes in motility, non motility associated parameters of sperms or the female germ line might affect fertility of *Ifitm1^tm1IEG/tm1IEG^* mutant mice, we analyzed the total number of pups born from matings between homozygous *Ifitm1* mutant males (n = 6) and either homozygous *Ifitm1* mutant females (n = 2) or wildtype C57BL/6J females (n = 4). Litter sizes from the homozygous matings were 7 and 10 pups, whereas the litter sizes from homozygous and wildtype matings were 5, 7, 8 and 9 pups. Since the average litter size of C57BL/6J generally is between 5 and 6 pups, our data provided no evidence for reduced fertility of homozygous *Ifitm1* mutant male and female mice.

### Analysis of Somites and Skeletons of Homozygous *Ifitm1* Mutant Animals

Knock-down experiments suggested that *Ifitm1* might be required for normal somitogenesis [35]. Thus, we analyzed the morphology of the somites and the neural tube of E9.5 embryos from heterozygous matings. There were no abnormalities in somite and neural tube formation evident in homozygous mutant embryos compared to wildtype littermates when embryos were inspected microscopically. Additionally, Alizarin red and Alcian blue stained skeletons of newborn homozygous mutant animals exhibited no abnormalities in bone and cartilage formation compared to wildtype littermates. Also, detailed inspection of cervical, thoracic, lumbar, sacral and tail vertebrae, limbs and skull, as well as of the size and morphology of dissected individual vertebrae, did not reveal any morphological abnormality. Thus, somite and vertebra formation appeared normal in homozygous *Ifitm1* knockout animals compared to wildtype littermates. At least upon microscopic inspection, all developing organs appeared morphologically normal in E9.5 *Ifitm1^tm1IEG/tm1IEG^* embryos.

### Determination of Leucocyte Numbers in *Ifitm1^tm1IEG/tm1IEG^* Animals

To examine whether the loss of *Ifitm1* function might affect the differentiation of cells of the immune system, we determined the proportions of different leucocyte subsets, such as granulocytes, monocytes, B-cells, T-cells, NK-cells, and NKT-cells in the blood of unchallenged male and female homozygous *Ifitm1* knockout mice compared to wildtype littermates by FACS analysis. Under baseline conditions there was no evidence for genotype specific differences between homozygous mutant and wildtype mice in the leucocyte patterns of the peripheral blood (n  = 11 for female *Ifitm1^tm1IEG/tm1IEG^* mice, n  = 9 for female wildtype mice, n  = 9 for male *Ifitm1^tm1IEG/tm1IEG^* mice, n  = 10 for male wildtype mice) (data not shown).

### Challenging Infection with *Listeria monocytogenes*

Previous in vitro studies suggested that *Ifitm1* might be an important mediator of the anti-proliferative action of IFNγ [27]. Proper IFNγ signaling is also required *in vivo* for a normal immune response to infection with *Listeria monocytogenes* (*L.m.*) [36]. Therefore, we investigated the role of *Ifitm1* upon challenge with *L.m*. Female homozygous *Ifitm1* mutant and wildtype mice were infected with approximately 50,000 wildtype *Listeria* (*L.m.*-wt) and on day 3 after infection the number of colony forming units (CFU) was determined in homogenates from infected spleen and liver to analyze primary susceptibility/resistance (Fig. S3, panel A). There were no significant differences in the number of CFU between homozygous knockout mice and wildtype littermates.

In order to analyze antigen-specific T-cell responses, *Ifitm1^tm1IEG/tm1IEG^* and *Ifitm1^wt/wt^* mice were also infected with approximately 5,000 *L.m.* expressing ovalbumin (*L.m.*-Ova). Seven days after infection, we analyzed the cytokine production of antigen-specific CD4^+^ and CD8^+^ T cells following short-term in vitro peptide re-stimulation (Fig. S3, panel B). In addition, after reinfection with a high dose of 250,000 *L.m.*-Ova, the ability of immunized mice to clear the pathogen and the cytokine producing CD4^+^ and CD8^+^ T cells were determined (Fig. S3, panel B). We found no significant differences between homozygous *Ifitm1* knockout mice and wildtype littermates in all these parameters. Thus, loss of *Ifitm1* does not affect the normal immune response to *L.m.* infection at least based on the parameters measured here. Based on these *L.m.* infection experiments, we found no evidence for a major dysfunction in downstream IFNγ signaling in *Ifitm1^tm1IEG/tm1IEG^* mice that would alter the pattern of resistance to infection.

### Genome-wide Expression Profiling of Adult Mouse Lungs

For a more comprehensive and less biased analysis of potential molecular phenotypes that could be induced by the loss of *Ifitm1* function, whole genome expression profiles were analyzed. We selected the adult lung because *lacZ* expression revealed clear and strong expression in this organ (see below). Affymetrix Gene ST 1.0 arrays were used to generate genome-wide expression profiles from male *Ifitm1* deficient (n = 5) and wildtype mice (n = 5) at the age of 120–150 days. As expected gene expression arrays detected a strong reduction of *Ifitm1* transcripts in lungs from homozygous *Ifitm1* knockout animals. To examine the annotated functions of regulated genes, we identified 123 regulated probe sets (corresponding to 110 genes) based on their statistically significant regulation (p<0.01) and searched for enriched GO (Gene Ontology) terms (Table 1). Significantly over-represented GO terms (p<0.01) associated with up-regulated genes in lungs of mutant mice were often related to inflammation and functions of the immune system, whereas down-regulated genes were frequently associated with cell cycle and metabolism-related GO terms (Table 1). Other upregulated genes in *Ifitm1* deficient mice were not associated with over-represented GO terms but are, for example, known to be expressed in immune cells (*Treml4*, 2.0-fold up) or in activated macrophages (*Erm4*, 2.0-fold up). The subtle but consistent down-regulation of *Ifitm2* (1.4-fold) might be due to the proximity of the chromosomal localization of the *Ifitm1* and *Ifitm2* gene loci. However, we did not observe any significant regulation of the *Ifitm3*, *Ifitm 5*, *Ifitm 6* or *Ifitm 7* transcripts in the comparison of mutant and wildtype lungs.

**Table 1:** Most significantly over-represented Gene Ontology (GO) terms among regulated genes in the comparative transcriptome analysis of *Ifitm1^tm1IEG/tm1IEG^* versus *Ifitm1^wt/wt^* lungs and list of regulated genes annotated with the respective GO terms.

GO Term	p-value	Gene symbol
**Upregulated genes**		
Defense response	1.49E-07	*Cnr2, Fcer1g, Fgr, Hck, Ly86, Myo1f, Scn3a, Tlr7, Tmem173, Tnfrsf1b*
Immune system process	2.49E-05	*Ccl6, Cd300e, Chrnb2, Fcer1g, ltgax, ltgb2, Ly86, Myo1f, Tlr7, Tmem173*
Tumor necrosis factorreceptor superfamily	5.92E-03	*Cnr2, Fcer1g, Gzma, lgh, ltgax, ltgb2*, *Tlr7, Tnfrsf1b, Tyrobp*
Inflammatory response	2.89E-05	*Cnr2, Fcer1g, Ly86, Scn3a, Tlr7, Tnfrsf1b*
Toll-like receptor	5.96E-03	*ltgax, Ly86, mmu-mir-223, Tlr7, Tm3m173, Tyrobp*
Leukocyte activation	9.44E-04	*Chrnb2, Fcer1g, ltgax, ltgb2, Myo1f*
T cell receptor CD3 complex	4.14E-03	*Fcer1g, Gzma, ltgax, ltgb2, Tyrobp*
Defense response to bacteria	2.10E-04	*Fcer1g, Fgr, Hck, Myo1f*
Chemotaxis	2.73E-03	*Ccl6, Fcer1g, ltgb2*
Other upregulated genes		*Emr4, Treml4, S100g, Igk, Ms4a4a, ENSMUST00000101072, Cybb, Ms4a6c, IghmAC38.205.12, I830127L07Rik, Sprr1a, Plac8, Retnlg, Ighv1-72, Gm7016*
**Downregulated genes**		
Mitochondrion	2.54E-03	*Acadvl, Alas1, Cox7a1, Cox8b, Cpt1b, Hspa1a, Mapk10*
Cellular lipid metabolic process	5.07E-03	*Acadvl, Akr1b7, Ces3, Cpt1b, Fabp3*
Regulation of cell cycle	3.13E-03	*Ccnd1, Hspa8, Rhob*
Other downregulated genes		*Ifitm1, Ifitm2, Ifi205, ENSMUST00000082857*

### Systemic Phenotyping in the German Mouse Clinic

To identify new mutant phenotypes 40 *Ifitm1^tm1IEG/tm1IEG^* mutant mice (20 males and 20 females) together with 39 wildtype littermates (20 males and 19 females) were comprehensively phenotyped in the primary screen of the German Mouse Clinic [37]. These screens did not reveal a distinct mutant phenotype, despite measuring about 320 phenotype parameters under standard conditions of mouse keeping. In particular, no significant differences were found in the dysmorphology, behavior, nociception, eye, and lung function screens. Modest but statistically significant changes between mutant and wildtype mice were identified in the neurological (modest reduction in rotarod latency), energy metabolism (trend towards increased mean oxygen consumption in female mutant mice), clinical chemistry (slight increase in specific hematological parameters of female mutant mice), immunology (slightly reduced frequencies of granulocytes and CD44 expressing T-cells in mutant mice), allergy (trend towards increased plasma IgE levels in mutant mice), steroid metabolism (slightly decreased corticosterone concentrations in female mutant mice, and high variance in testosterone concentrations in male mutant mice), and pathology (slight increase in liver weights normalized to body weights) screens. These differences between mutant and wildtype animals were subtle and further investigations are required to clarify whether these findings depend on *Ifitm1* gene function. The complete phenotype dataset is publically available from the EuroPhenome Mouse Phenotyping Resource [38], [39] and the phenotyping protocols of the primary screen of the German Mouse Clinic were previously published [40].

### *LacZ* Reporter Gene Expression

Since our reassessment of previously predicted *Ifitm1* functions did not provide evidence for a clear functional requirement of this gene *in vivo*, we comprehensively analyzed the *lacZ* expression pattern of heterozygous *Ifitm1^tm1IEG/wt^* embryos using the highly sensitive X-gal staining. The comparison of whole embryo RNA in situ hybridization patterns using a probe specific for the *Ifitm1* mRNA versus the *lacZ* reporter gene expression pattern in *Ifitm1^tm1IEG/+^* E9.5 embryos demonstrated that the reporter gene expression faithfully reproduces the endogenous *Ifitm1* expression pattern (Fig. S4). However, detection of gene expression using the X-gal staining was more sensitive than the RNA in situ detection method. In particular, staining in the paraxial mesoderm and the ventral brain was more intense using the X-gal staining as compared to the RNA in situ detection (Fig. S4). Since the *Ifitm1* gene expression pattern following E7.5 during embryogenesis has not yet been described, we comprehensively analyzed the expression pattern in embryos from E8.5 to E14.5.

### *Ifitm1* Expression from E8.5 to E11.5

**Figure 2:**
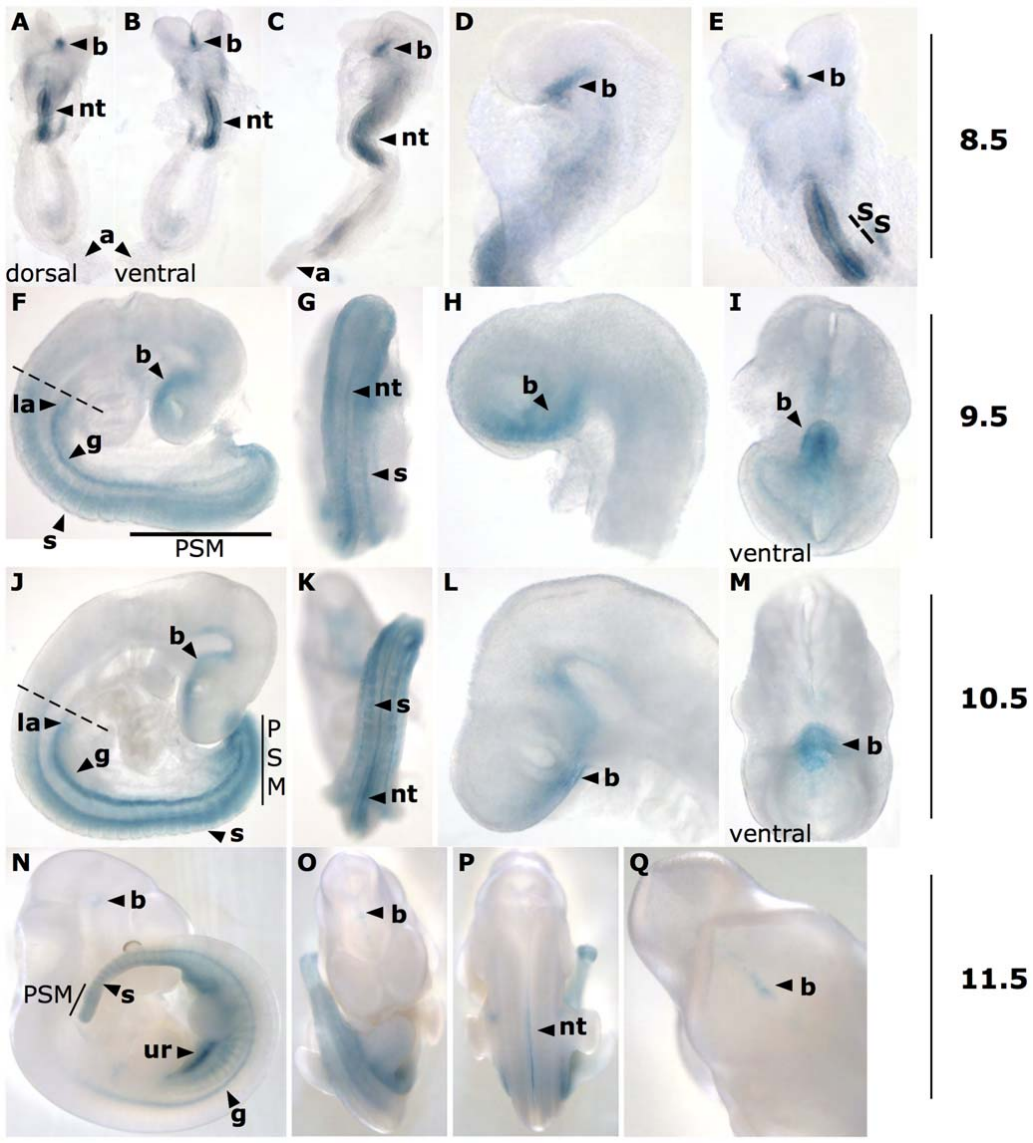
*LacZ* expression pattern in *Ifitm1^tm1IEG/wt^* embryos from E8.5 to E11.5. (**A – E**) At E8.5 strong expression was found in the future brain and the neural tube. Weak expression was detected in the allantois. (**F – I**) At E9.5 *lacZ* expression was detected in the future brain, the primordial gut, the somites and the presomitic mesoderm. The anlage of the lung was the anterior boundary of reporter gene expression in the trunk region (see dashed line). (**J – M**) At E10.5 the reporter gene expression pattern was similar to the one at E9.5. The expression in the ventral brain was extended along the anterior-posterior axis. (**N – Q**) At E11.5 *lacZ* expression decreased in the PSM and in somites. Weak expression was detected in the brain and a new expression domain developed in the urogenital ridge. Abbreviations: a – allantois, b – brain, g – primordial gut, la – anlage of the lung, nt – neural tube, PSM – presomitic mesoderm, s – somite, ur – urogenital ridge.

At E8.5 strong *lacZ* expression was detected in a restricted, ventral region of the developing brain and in the developing neural tube. Weak staining of the reporter gene was evident in single cells of the allantois (Fig. 2, A–E).

At E9.5 expression staining in the posterior mesoderm was strongly increased as compared to E8.5. *LacZ* was clearly expressed in the presomitic mesoderm (PSM) and the recently formed somites as well as in the neural tube and the developing gut. The anterior boundary of reporter gene expression in somites, neural tube and gut was approximately at the level of the anlage of the developing lung. *LacZ* expression was also found in a restricted, ventral region of the developing brain at this developmental stage (Fig. 2, F–I).

At E10.5 the reporter gene expression pattern was similar to the one at E9.5, albeit even stronger in the PSM, somites, the gut and the neural tube with an anterior boundary at the level of the anlage of the lung. The expression in the developing brain at E10.5 was again ventrally restricted but extended further along the anterior-posterior axis as compared to E9.5 (Fig. 2, J–M).

At E11.5 the expression pattern changed considerably. The intensity of beta-galactosidase staining in the PSM, the somites, the neural tube and the ventral region of the developing brain strongly decreased as compared to the earlier developmental stages. A new expression domain in the urogenital ridge was evident (Fig. 2, N–Q).

### *Ifitm1* Expression from E12.5 to E14.5

**Figure 3:**
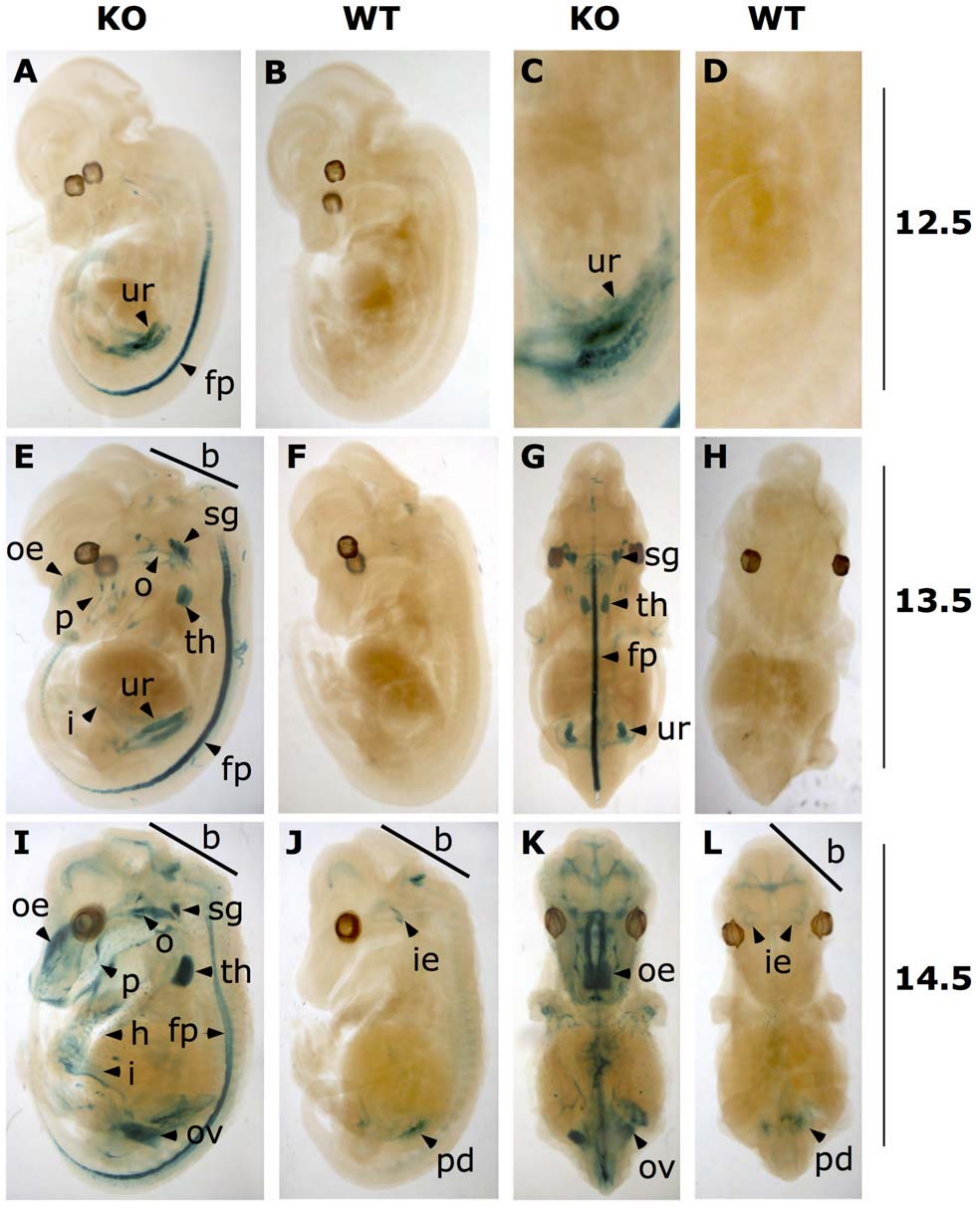
*LacZ* expression pattern in E12.5 to E14.5 *Ifitm1^tm1IEG/wt^* mouse embryos. **A–D**: E12.5: *lacZ* was highly expressed in the floor plate of the neural tube and the urogenital ridge. **E – H**: E13.5: several new embryonic *lacZ* expression domains appeared in the brain, the intestine, the mucosa of the oropharynx, the olfactory epithelium, the papilla of the tongue, the sympathetic ganglion and the thymus. Background staining due to unspecific X-Gal staining was found in brain. **I – L**: E14.5: additional expression domains were detected in the primordia of hair follicles as well as in the ovary. Background staining in wildtype littermates was found in the brain, the inner ear, and the paramesonephric duct. b – brain, fp – floor plate, h – primordium of hair follicle, i – intestine, ie – inner ear, o – oropharynx, oe – olfactory epithelium, ov – ovary, p – papilla of the tongue, pd - paramesonephric duct, sg – sympathetic ganglion, th – thymus, ur – urogenital ridge.

At E12.5 staining for *lacZ* expression in the paraxial mesoderm decreased markedly such that it was barely detectable. In contrast, strong expression was observed in the cells of the floor plate of the neural tube and the urogenital ridge. The anterior boundary of reporter gene expression in the developing gut, the paraxial mesoderm and the neural tube at the level of the anlage of the lung now appeared more diffuse than at earlier stages (Fig. 3, A and C). No background staining was detected in wildtype embryos of this developmental stage (Fig. 3, B and D).

**Figure 4:**
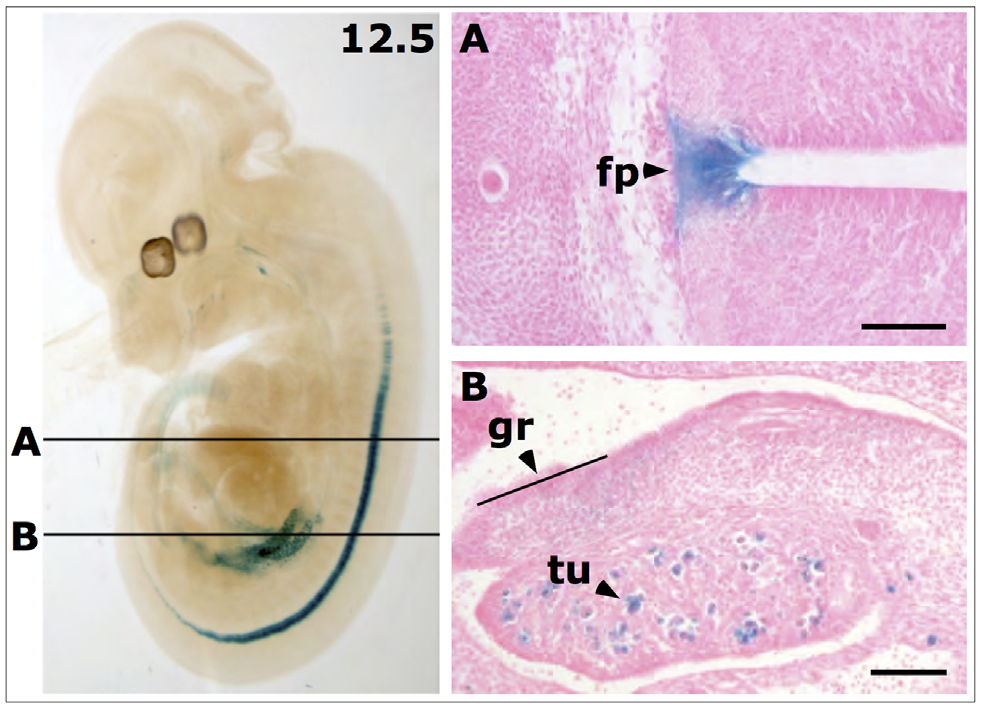
Histological sections of *LacZ* stained *Ifitm1^tm1IEG/wt^* mouse embryo at E12.5. Lines in the bleached whole embryo (left panel) indicate planes of histological sections in panels **A** and **B** on the right. (**A**) *LacZ* expression was found in cells of the floor plate (fp) of the neural tube. (**B**) Gene expressions in cells of the genital ridge (gr) is weak and strong in the tubuli of the metanephros (tu). Scale bars indicate 100 µm.

Histological sections of stained embryos at E12.5 revealed strong expression in the tubuli of the metanephros. Expression in the genital ridge now was very faint and diffuse. Strong expression in the cells of the floor plate was detected (Fig. 4, A–B).

**Figure 5:**
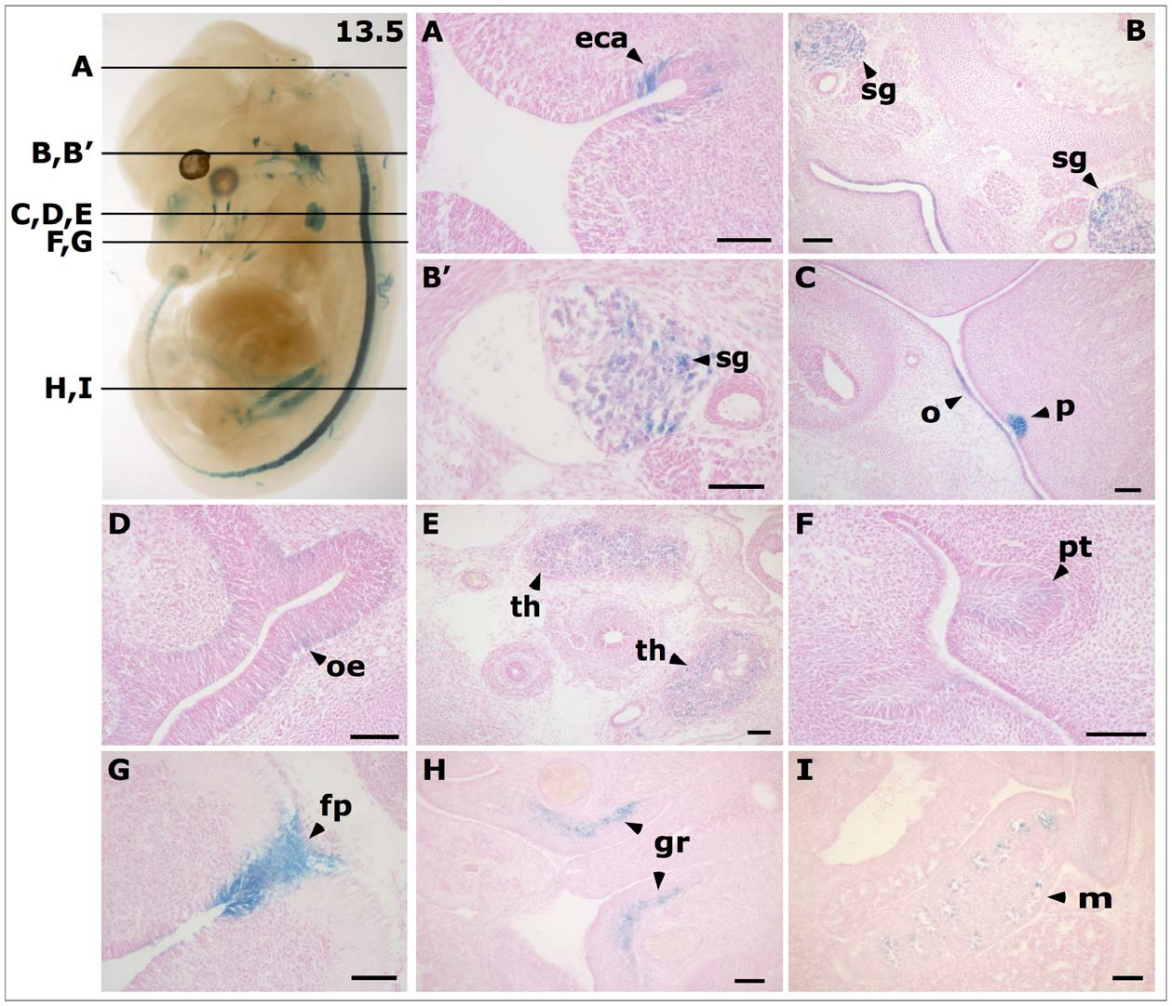
Histological sections of *LacZ* stained *Ifitm1^tm1IEG/wt^* mouse embryo at E13.5. Lines in the bleached whole embryo (panel at the top left) indicate planes of histological sections (**A** to **E**). (**A**) *LacZ* expression was found in the epithelium of the cerebral aqueduct (eca), (**B** and **B’**) the sympathetic ganglion (sg), (**C**) in the papillae of the tongue (p) and the epithelium of the oropharynx (o), (**D**) the olfactory epithelium (oe), (**E**) the thymus (th), (**F**) primordia of the teeth (pt), (**G**) cells of the floor plate (fp) of the neural tube, (**H**) the genital ridge (gr) and the (**I**) metanephros (m). Scale bars indicate 100 µm.

At E13.5 *lacZ* reporter gene expression occurred in additional developing organs as compared to the pattern at E12.5 (Fig. 3, E and G). It was expressed in the brain, cells of the sympathetic ganglion, the mucosa of the oropharynx, the olfactory epithelium, the floor plate, the papilla of the tongue, primordial of the teeth, the thymus, the genital ridge, the intestine, the metanephros and the inner side of limbs. Background staining in wildtype embryos appeared only in the choroid plexus of the brain at E13.5 (Fig. 3, F and H). In histological sections, we could assign the *lacZ* expression in the brain to the epithelium of the cerebral aqueduct (Fig. 5, A). The outer strong expression in the sympathetic ganglion was not distributed evenly in all cells (Fig. 5, B – B’). The epithelium of the oropharynx was another epithelial structure that showed *lacZ* expression in histological sections. Expression was also found in the papillae of the tongue (Fig. 5, C). Histological sections of the olfactory epithelium showed moderate X-Gal staining at the basal level of the epithelium (Fig. 5, D). Interestingly, sections of the stained thymus revealed more prominent *lacZ* expression in the cortex than in the medulla (Fig. 5, E). The expression in the primordial teeth was clearly visible in histological sections but weaker compared to the strong X-Gal staining in the cells of the floor plate (Fig. 5, F–G). X-Gal staining in the genital ridge was found in an inner cell layer (Fig. 5, H). Expression in the metanephros could be assigned to the tubuli (Fig. 5, I).

**Figure 6:**
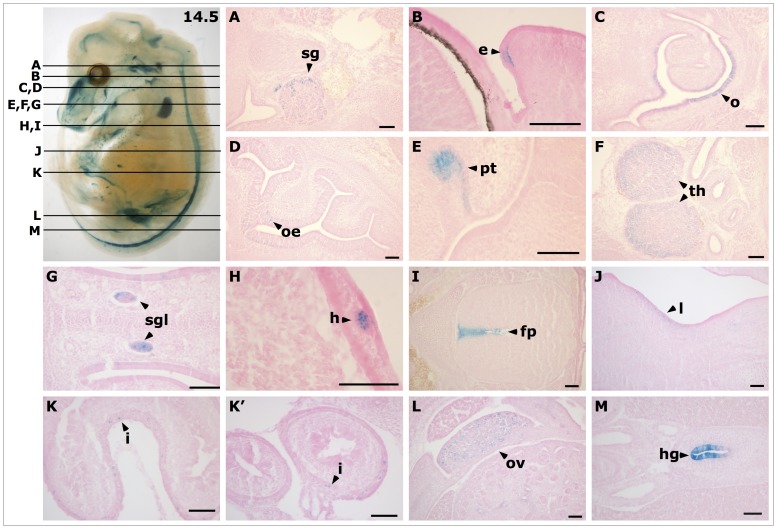
Histological sections of *LacZ* stained mouse *Ifitm1^tm1IEG/wt^* embryo at E14.5. Lines in the bleached whole embryo (panel at the op left) indicate planes of histological sections (**A** to **M**). (**A**) *LacZ* expression was found in sympathetic ganglia (sg), (**B**) the eyelids (e), (**C**) the epithelium of the oropharynx (o), (**D**) the olfactory epithelium (oe), (**E**) primordia of the teeth (pt), (**F**) the thymus (th), (**G**) the serous glands associated with the nasal septum, (**H**) primordia of hair follicles (h), (**I**) in cells of the floor plate (fp) of the neural tube, (**J**) mesenchymal cells at the inner side of the limbs (l), (**K** and **K’**) cells of the outer wall of the intestine (i), (**L**) cells of the ovary (ov), and (**M**) in cells of the hindgut (hg). Scale bars indicate 100 µm.

At E14.5 reporter gene expression in the whole embryo was again strong and new expression domains were evident as compared to the embryos at earlier stages. In addition to the expression domains at E13.5, cells of the eyelid, the primordia of hair follicles all over the embryo and the ovary expressed *lacZ* (Fig. 3, I and K). The background staining in wildtype embryos at E14.5 was found in additional organs. It was now detected in various regions of the brain, in the inner ear as well as in paramesonephric tubes (Fig. 3, J and L). In histological sections of E14.5 embryos, *lacZ* expression was evident in sympathetic ganglia (Fig. 6, A) similar to the expression at E13.5. Expression in the eyelid was found in epithelial cells that might later have secretory function (Fig. 6, B). *LacZ* expression was predominantly located at the basal side of these cells. Expression in the primordial teeth became more prominent and extended throughout the developing teeth primordia (Fig. 6, E), and there was a more prominent staining in the cortex than in the medulla of the developing thymus (Fig. 6, F). The histological sections revealed a new domain of expression at E14.5 in the tubuli of serous glands associated with the nasal septum (Fig. 6, G). The primordial hair follicles showed a spotted expression within single cells (Fig. 6, H). The externally visible *lacZ* staining on the inner side of the limbs was located under the dermis in mesenchymal cells (Fig. 6, J). Reporter gene expression in the intestine was located to single cells in the outer epithelium of the intestinal wall at E14.5 (Fig. 6, K, K’). The latter expression in the intestine was also detected at E13.5, but was too faint to be seen in the histological sections at that stage. Finally, *lacZ* expression was found in cells of the developing ovary but not in the kidney (Fig. 6, L) and was strong throughout the epithelium of the hindgut (Fig. 6, M).

### *Ifitm1*/IFITM1 Expression in Mouse and Human Adult Organs and in Human Lung Carcinomas

**Figure 7:**
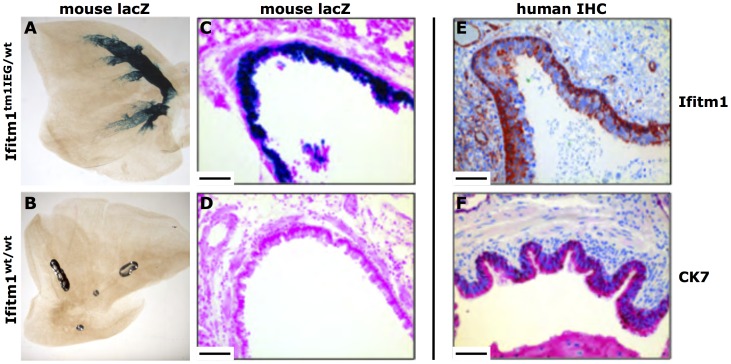
*Ifitm1*/IFITM1 expression in mouse and human lung. (**A** and **B**) Detection of *Ifitm1* expression in adult mouse lung by whole organ X-Gal staining. *LacZ* expression was detected in the bronchia of the lung of *Ifitm1^tm1IEG/wt^* adult mice (**A**) but not in wildtype lungs (**B**). (**C** and **D**) Subsequent Histological sections of stained *Ifitm1^tm1IEG/wt^* mouse lungs revealed *lacZ* expression in the cells of the bronchial epithelium of *Ifitm1^tm1IEG/wt^* mice. (**E**) By immunohistochemistry on human adult lung tissue, IFITM1 expression was detected in the columnar- and in the basal cells of the bronchial epithelium. (**F**) Immunohistochemical detection of CYTOKERATIN*-7* (CK7) was used to identify columnar cells of the bronchial epithelium. Scale bars indicate 100 µm.

**Figure 8:**
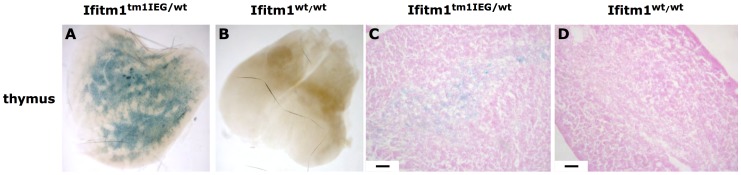
Reporter gene expression in thymus from adult *Ifitm1^tm1IEG/wt^* and wildtype mice. (**A** and **B**) Whole organ X-Gal staining of the thymus of heterozygous knockout animals exhibited *lacZ* expression whereas thymus of wildtype littermate mice did not stain for X-Gal. (**C** and **D**) Histological sections of stained thymus revealed cells of the medulla expressing the *lacZ* reporter gene in *Ifitm1^tm1IEG/wt^* mice (**C**) but not in thymus from wildtype littermate mice (**D**). Scale bars indicate 100 µm.

In adult *Ifitm1^tm1IEG/wt^* mice (90–120 days old) we analyzed *lacZ* expression in dissected brain, intestine, kidney, liver, lung, ovary, pancreas, spleen, tongue and thymus using X-Gal staining. We found reproducible and strong staining for *lacZ* expression in the lung (Fig. 7) and the thymus (Fig. 8).

In the adult thymus *Ifitm1* expression was found throughout the entire organ in distinct cells of *Ifitm1^tm1IEG/wt^* mice but not in wildtype mice (Fig. 8, A and B). Histological sections of stained thymus from *Ifitm1^tm1IEG/wt^* mice revealed *lacZ* expression in cells of the medulla. No reporter gene expression was found in the cortex of the thymus (Fig. 8, C and D).

Macroscopically, *lacZ* expression was clearly visible also in the bronchia of the lung from *Ifitm1^tm1IEG/wt^* animals but not in wildtype mice (Fig. 7, A and B). Histological sections of the stained adult lung revealed *lacZ* reporter gene expression in the cells of the bronchial epithelium in mutant but not in wildtype mice (Fig. 7, C and D).

**Figure 9:**
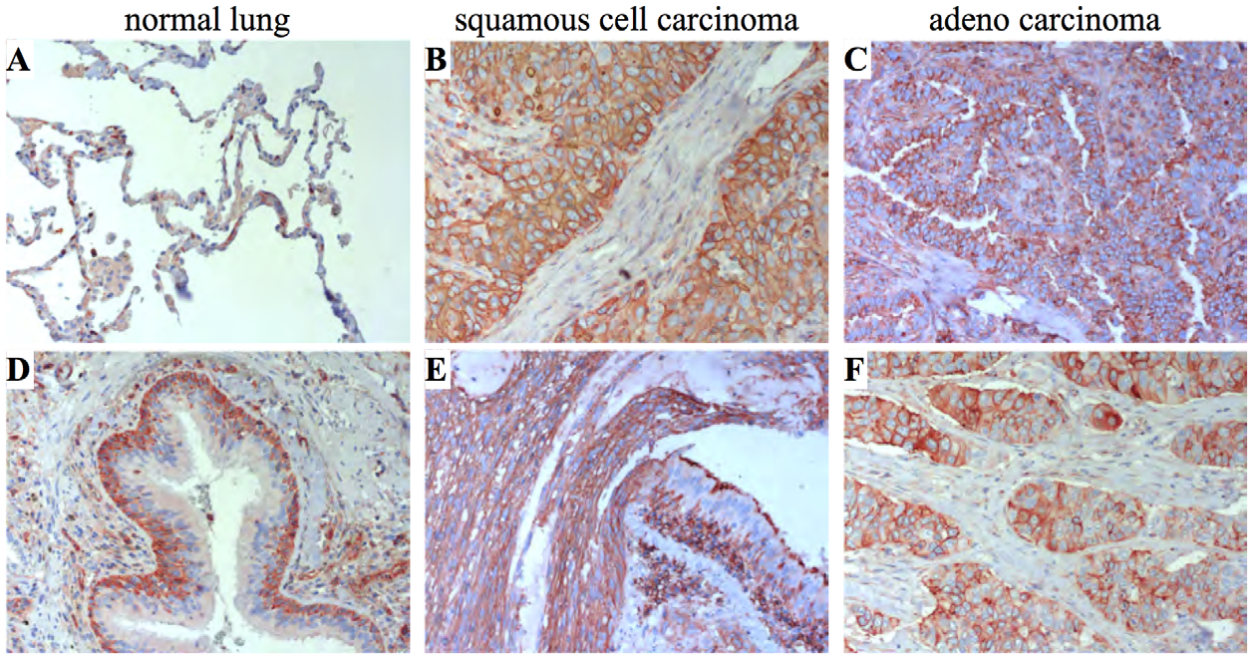
IFITM1 immunohistochemistry on human lung tissue (40x). (**A** and **D**) IFITM1 is expressed in non-neoplastic alveolar and bronchial epithelia. (**B** and **E**) The IFITM1 gene is highly overexpressed in squamous cell carcinomas of the human lung and (**C** and **F**) in adenocarcinomas of the human lung.

We analyzed IFITM1 expression in human lung tissue by immunohistochemistry and found IFITM1 expression in columnar- and basal cells of the bronchial epithelium (Fig. 7, E). In contrast to the expression in mice, human IFITM1 expression was predominantly detected in the basal cell layer rather than in the predominantly CYTOKERATIN-7 (CK7) expressing columnar cells (Fig. 7, F). IFITM1 expression was detected in non-neoplastic alveolar and bronchial epithelium of the normal human lung (Fig. 9, A, D). Interestingly, we found strong IFITM1 overexpression in the tumor epithelia cells of human squamous cell carcinomas (Fig. 9 B and C) as well as in adenocarcinomas of NSCLC patient samples (Fig. 9, E and F).

## Discussion

In previous studies several functions for the *Ifitm1* gene have been postulated, but only its requirement for primordial germ cell (PGC) development and migration has been studied in detail [1], [2], [28]–[33]. Other suggested functions of the *Iftim1* gene include a role for this gene during somitogenesis [35], and in INF signaling and immune response [5], [14], [20], [22], [24], [27], [36], [41], [42]. However, these functional associations for *Ifitm1* were, so far, inferred from coincidence of gene expression data, the sequence based identification of cis-regulatory response elements, or in vitro and knock-down studies [1], [35]. The only *in vivo* study of *Ifitm* gene functions [1] has analyzed the requirement of *Ifitm3* and of the entire *Ifitm* gene cluster in mouse models. The phenotype analysis focused on the suspected functional requirement of *Ifitm* genes for PGC formation and migration. Other potential phenotypes were not investigated in detail. Therefore, the true functional role of the *Ifitm1* gene *in vivo* has remained elusive, which asked for the reassessment of *Ifitm1* functions. Here, we establish a new knockin mouse model by replacing the entire coding region of the *Ifitm1* gene with a *lacZ* reporter gene to systematically analyze the suggested functions of the *Ifitm1* gene *in vivo*. We confirm the previous notion that *Ifitm1* is not required for normal PGC migration and male and female fertility. In addition, we find that functional *Ifitm1* is not necessary for normal somite formation and development as well as for a normal immune response even under conditions of *Listeria* infection. To provide a rational basis for the reassessment of *Ifitm1* function, we have undertaken a comprehensive analysis of the complex and dynamic *Ifitm1* gene expression pattern in embryonic and adult tissues. Furthermore, our data underline the importance of functional *in vivo* studies since most predicted functions of *Ifitm1* were not confirmed in our study.

### *Ifitm1* Mutant Mice have Normal Male and Female Fertility

*Ifitm* genes were suggested to have key functions in the early phase of germ cell development [1], [2], [28], [30]–[33]. Unexpectedly, the targeted deletion of the whole *Ifitm* gene cluster as well as the knockout of the *Ifitm3* gene in mouse models revealed no phenotypic alteration in PGC development and migration, questioning the suspected gene function [1]. To further substantiate these findings, we present here the first detailed analysis on the requirement specifically of *Ifitm1* for sperm cell development. We used a sperm motility assay to characterize the sperm quality of adult male mice deficient for *Ifitm1*. No significant differences were detected between sperms from *Ifitm1^tm1IEG/tm1IEG^* and *Ifitm1^wt/wt^* males in all parameters of the sperm motility assay. This also supports the detailed analysis of Lange *et al.* 2008 in which no changes in PGC development were evident in mice carrying the full *Ifitm* cluster deletion. Our breeding results confirmed the results from the sperm motility assay as *Ifitm1^tm1IEG/tm1IEG^* male as well as female mice had normal fertility. However, we cannot fully exclude the possibility that the fertility of *Ifitm1^tm1IEG/tm1IEG^* mutant mice under particularly challenging environmental or experimental conditions (such as during in vitro fertilization) could be affected.

### *Ifitm1* Mutant Mice Develop Normal Somites and Vertebrae

The postulated requirement of *Ifitm1* for somitogenesis was deduced from the phenotype of an *Ifitm1* targeted knock-down using RNA interference (RNAi), which resulted in smaller and irregular somites in mouse embryos [35]. To reassess the necessity of *Ifitm1* for normal somite development, we analyzed somite formation and development of homozygous knockout E9.5 embryos in comparison to wildtype littermates. We found no abnormalities in the shape and size of somites as well as no abnormalities in the formation of the neural tube. In addition, we carefully examined the morphology of skeletons from homozygous *Ifitm1* mutant mice and compared them to skeletons of wildtype littermates as an indicator for correct somite differentiation. We found no phenotypic alterations in the skeletons of *Ifitm1^tm1IEG/tm1IEG^* newborn mice regarding the vertebral pattern and vertebra morphology, the formation and number of ribs, formation of intervertebral discs, and morphology of the skull and bones of the limbs. This is in contrast to the results of the knockdown of *Ifitm1* using RNA interference, which provided evidence for a somite phenotype [35]. In the latter study, somites of knock-down embryos were irregular and smaller than wildtype somites and knock-down embryos showed a kinked neural tube. None of these knock-down phenotypes were observed in our homozygous knockout embryos. We suspect that this difference between *Ifitm1* knock-down and knockout mice might be due to off-target interactions of the RNAi with mRNAs other than the *Ifitm1* transcript, again emphasizing the importance of targeted *in vivo* studies for revealing true gene functional requirements.

### *Ifitm1* Mutant Mice have Normal Leucocyte Populations and Normal IFNγ Response Following *Listeria monocytogenes* Challenge

Genes of the *Ifitm* family have frequently been implicated in IFN signaling and tumor formation [6], [7], [10]–[17], [19]–[27], [41], [43]–[45]. In particular, it was hypothesized that signaling of type I and type II IFN might act through *Ifitm1* [7]. Furthermore, *Ifitm1* was also regulated in different forms of tumors [9]–[11], [13], [14], [24], [46]. In contrast, no immunological phenotype was found in the knockout of the whole *Ifitm* gene cluster [1]. To further substantiate these results, we analyzed the leucocyte populations in peripheral blood and IFNγ mediated immune response following *L.m.* infection in the *Ifitm1^tm1IEG/tm1IEG^* and control littermates. We chose *L.m.* for these experiments because protective immune responses to infection act substantially via IFNγ signaling [27]. No statistically significant differences were observed between *Ifitm1* mutant and wildtype animals. We provide the first report of a detailed immunological analysis of *Ifitm1* deficient mice towards an infection challenge.

As all the former results on the role of *Ifitm1* for IFN signaling were generated *in vitro* or deduced from coincidental data, our results again demonstrate the importance of targeted *in vivo* studies. However, we cannot exclude the possibility that under other challenge conditions *Ifitm1* deficient mice might reveal an involvement of this gene in immune function.

### Gene Expression Signature in Adult Lungs

We chose genome wide expression profiling as an unbiased approach to analyze the molecular phenotype on the mRNA level in adult lungs of *Ifitm1^tm1IEG/tm1IEG^* mutant mice. Our results confirmed the loss of *Ifitm1* transcripts in knockout animals, whereas other genes in the *Ifitm* cluster were not significantly regulated at least in this organ (Table 1). Therefore, we consider it unlikely that the lack of a detected mutant phenotype was caused by compensatory regulation of other *Ifitm* genes. *Ifitm2* showed a modest down-regulation in lungs of homozygous mutant animals of about 1.4-fold. *Ifitm2* is a direct neighboring gene of *Ifitm1*. We therefore suspect that the observed down-regulation of *Ifitm2* might be due to the vicinity of the targeted *Ifitm1* locus and the presence of transgenic sequences that might affect the expression of *Ifitm2*.

Interestingly, the up-regulated genes in mutant animals were predominantly involved in inflammatory processes or associated with an immune response (Table 1). Further immunological studies on *Ifitm1* knockout mice, possibly by exposing these mouse models to different envirotypes, might be required to characterize the role of *Ifitm1* in the potential of the lung to respond to an immunological challenge or infection.

### *Ifitm1* is Expressed in Epithelia, Neural Tissues and Thymus during Development and in Adult Tissues

Our detailed *in vivo* analysis of *Ifitm1* functions provides clear data that strongly questions the previously suspected functions of this gene. The functional requirement of *Ifitm1* in mammals thus remains enigmatic. To begin a more systematic approach to reveal (the) function(s) of *Ifitm1*, we have undertaken the first detailed and sensitive analysis of *Ifitm1* expression patterns throughout embryonic development from E8.5 to E14.5 and in adult tissues. The discovery of new expression domains will probably be trendsetting for the identification of the *Ifitm1* gene functions.

We found a complex and dynamic *Ifitm1* expression pattern throughout developmental stages and restricted expression domains in adult organs. Expression of the reporter gene from the targeted *Ifitm1* gene locus was found in tissues derived from all three embryonic germ layers. During mouse embryogenesis we found that the *Ifitm1* reporter gene is frequently expressed in several epithelial structures, such as the tubuli of the metanephros, the oropharynx, the olfactory epithelium, the developing tooth primordia, the developing eyelids, the walls of the embryonic intestine, and the glands associated with the nasal septum. In adult mice it was expressed in the bronchial epithelial cells. In developing neuronal tissues we found the reporter gene expressed in the floor plate of the neural tube and sympathetic ganglia. In addition we found *lacZ* expression in the developing thymus, in fibroblasts of the limbs and in gonads.

The *Ifitm1* expression in mucosa cells of the bronchial epithelium of adult mice is of particular interest in the context of lung cancer, which is responsible for the most frequent cancer associated deaths worldwide. The bronchial epithelial cells represent the epithelial origin for human small- and non-small lung cancers. Accordingly, we find expression of the human IFITM1 gene especially in the normal human bronchial epithelium (Fig. 7, E and Fig. 9 A and D) as well as increased expression in epithelial derived human lung carcinomas based on immunohistochemistry (Fig. 9, B, C and E, F). Since *IFITM1* has been proposed as a negative regulator of cell proliferation, it might have a key role in tumor formation [27]. This hypothesis is also supported by studies that identified lung cancer related genes in the chromosome band 11p15.5 in man where *IFITM1* is located [47]. Loss- and gain-of *Ifitm1* function experiments in lung cancer mouse models might help understanding the role of *Ifitm1* in lung cancer development.

### Conclusion

Based on our findings the suspected functions for *Ifitm1* as well as other genes of the *Ifitm* gene family could not be confirmed by our targeted *in vivo* studies. Here, the comprehensive description of *Ifitm1* gene expression patterns in mouse embryos and adult organs now provides a basis for more systematic and targeted approaches to uncover unique *Ifitm1* functions *in vivo*. Especially the role of *Ifitm1* for lung cancer development and treatment might be in the focus of interest. Revealing these functions might also require challenging the *Ifitm^tm1IEG^* mutant mice, mating them to other mutant lines and exposing them to additional envirotypes [48].

## Materials and Methods

### Ethics Statement

All animal experiments were approved by the Government of Upper Bavaria, Germany (Regierung von Oberbayern, Deutschland), and conducted according to this approval and to the German Law for Animal Protection (Deutsches Tierschutzgesetz).

Human formalin fixed paraffin embedded (FFPE) tissue samples were kindly provided by the Institute of Clinical Pathology at the Medical University of Vienna (MUW). All experiments with human tissue samples were approved by the ethical committee of the MUW, in compliance with Austrian legislation after receipt of informed patient written consent and in accordance with the declaration of Helsinki.

### *Ifitm1* Targeting Vector Design

For the generation of *Ifitm1* knockout mutant mice we designed a targeting vector replacing the entire coding region of the *Ifitm1* gene by a *lacZ* reporter gene (Fig. 1). We designed a fusion PCR to insert the *lacZ* reporter gene (pSV-β-Galactosidase Control Vector, Promega) in frame with the transcription start of the *Ifitm1* gene (f – AGC CGA GAG **ATG GGG**GAT CCC GTC GTT TTA, r – GTT GCA CCA CAG ATG AAA CG). The *lacZ* reporter gene was followed by a bGHpA site and a neomycin selection cassette flanked by loxP sites under the control of a PGK/EM7 promoter for positive selection. For negative selection a diphtheria toxin (DT) cassette (pKO SelectDT, Stratagene) was introduced into the bacterial backbone of the pBluescript-II-KS+ vector. Prior to electroporation in mouse ES cells, the targeting vector was linearized with the AflII restriction enzyme (MBI Fermentas, Germany) and ethanol precipitated.

### ES Cell Targeting and PCR Genotyping

E14 embryonic stem (ES) cells were targeted with the Amaxa nucleofector system in Amaxa mouse ES cell nucleofector solution with program A-23. 3×10^6^ cells were targeted using 1–10 µg of linearized targeting vector DNA, dispensed on feeder plates and selected with 0.4 mg/ml G418 (Gibco, Germany). Colonies were picked at day 10 of selection and grown and split in 96 well plates. Cells from confluent 96 well plates were used for DNA preparation and genotyping. After two changes of phosphate buffered saline (PBS), 50 µl of Kawasaki buffer (20 mM TrisHCl pH 8.3, 25 mM KCl, 1.5 mM MgCl_2_, 0.5% Tween 20) containing 1 µl of proteinase K (20 mg/ml) were dispensed on each well and incubated at 60°C over night. Proteinase K was heat inactivated at 96°C for 30 minutes and samples were used directly in PCR reaction. The ES cells were genotyped with the primers ES-PCR-f1– GAC GTA AAC TCC TCT TCA GAC C and ES-PCR-r1– GGG AGA GAG TAG AGA AGT AAA GGC (see ES PCR in Fig. 1 and panel A in Fig. S1).

### Southern Blot Analysis

Clones identified as targeted by PCR genotyping were subsequently grown for harvesting sufficient genomic DNA for Southern blot analysis. DNA was isolated from ES cells (and later from mouse tails) by standard procedures and 15 µg of genomic DNA were digested with PvuII restriction enzyme (MBI Fermentas, Germany) over night. After gel-electrophoresis, digested genomic DNA was blotted to a Hybond-N+ membrane (GE Healthcare Life Science, Germany) by neutral transfer. Hybridization of Southern blots was done with probes labeled with an AlkPhos direct labeling and detection system (GE Healthcare Life Science, Germany) according to supplier’s instructions (see panel B in Fig. S1). The DNA hybridization probe (Fig. 1) was amplified from genomic DNA with the primers sp-f2– CAA ACC CTA GAC TTA GTC CTG ACC and sp-r2– GTA CTC TTC TGA AGG CAC CTC TAC.

### Animal Breeding and Genotyping

Targeted ES cell clones with homologous recombination of the targeting vector were used for injection into C57BL/6J blastocysts and embryo transfer into CD1 foster females. Male chimeras were bred to C57BL/6J^Cre/+^ females to obtain germ line transmission and to remove the positive selection cassette from the genome. The offspring of these breedings was screened for the *Ifitm1* floxed knockout allele (*Ifitm1^tm1IEG^*) by PCR (f3– GGG AGG ATT GGG AAG ACA ATA G, r3– GTA CTG TAC AGC CAA GGT TAG G) as well as for the *Ifitm1* wildtype allele (f4– GGT AGT CGT GAG TGT GGT AAA G, r3). Heterozygous *Ifitm^tm1IEG/+^* mutant siblings were mated to establish the stable *Ifitm1* knockout line.

### Beta-galactosidase Staining in Embryos and Adult Tissues

Embryos from timed pregnancies were dissected in PBS and fixed in 4% PFA for 30–45 minutes at room temperature depending on the developmental stage. After fixation embryos were washed in permeability buffer (2 mM MgCl_2_, 0.2% NP-40, 0.01% SDS in PBS) for 45 minutes at room temperature and subsequently incubated in X-gal staining buffer (10 mM potassium ferricyanide crystalline, 10 mM potassium ferricyanide trihydrate, 2 mM MgCl_2_, 0.02% NP-40, 0.01% sodium deoxycholate, 1∶40 X-gal in N, N dimethylformamide in PBS pH 8.2) over night at 37°C. After staining embryos were washed in PBS and post-fixed in 4% PFA over night at 4°C. Embryos were inspected with a Leica MZ16F microscope. Photos were taken with a Leica DCF320 camera.

For histological sections embryos at E12.5 to E14.5 were cryopreserved in 30% sucrose over night, cryo-embedded in OCT and stored at −20°C over night. Embryos were sectioned at 20 µm and −25°C using a Leica cryotome and transferred onto histological glass slides. Sections were fixed in 4% PFA for 2 minutes, washed twice in PBS for 5 minutes and counterstained using nuclear fast red (Sigma-Aldrich, N3020) for 5 minutes. Sections were then washed in distilled water and dehydrated in an ascending ethanol series for 5 minutes each, xylene treated and mounted using the Roti Histo Kit. Adult organs were stained and sectioned in the same way as embryos. Stained histological sections were inspected with a Zeiss Axiovision microscope.

### Histological and Immunohistochemical Analysis of Human Lung Samples

Histological analyses of human lung samples were performed on formalin-fixed and paraffin-embedded tissue. H&E staining was performed on 4 µm thick sections according to the standard procedures. The slides were mounted with DAPI in Vectashield (Vector Laboratories). Primary antibodies for IHC were human *Ifitm1* (Sigma-Aldrich, HPA004810) and human CK7 (DAKO, M7018). Primary antibodies were detected by the Immunoperoxidase method using the IDetect Super Stain System HRP (ID labs Biotechnology). Signals were amplified using 3-amino-9-ethylcarbazole (ID labs Biotechnology) under visual control. Afterwards, the sections were counterstained with Mayer’s hematoxylin. The images were captured with a Zeiss AxioImager Z1 microscope using a PixeLINK camera with the corresponding acquisition software. IHC stainings were imaged on a Zeiss stereomicroscope with camera.

### Sperm Quality and Motility Assay

The sperm quality was measured as described previously [49]. Briefly, fresh epididymal sperm was collected from 6 months old males in 500 µl HTF medium [50]. Sperms were taken from the epididymis and spermatic duct and incubated in the medium at 37°C for 5 min. 10 µl of the sperm suspension from each male was transferred to a 500 µl drop of HTF medium and incubated again for 10 min at 37°C and 5% CO_2_. 15 µl of this diluted sperm suspension were measured using an IVOS Sperm Analyzer (Version 12.1c; Hamilton Thorne Research). The selected parameters were 60 frames per second (30 frames total), minimum contrast 70, and minimum cell size 1 pixel. The following 9 cell track parameters were evaluated: percentage of motile cells, percentage of progressive cells (VAP>60 µm/s and STR>50%), velocity of average path (VAP), velocity of straight line (VSL), velocity of the curvilinear path (VCL), straightness (STR = VSL/VAP), linearity (LIN = VSL/VCL), average lateral head displacement (ALH) and beat cross frequency (BCF).

### Analysis of Leucocytes

Blood of 12 weeks old male and female mice was isolated from the orbital plexus. After centrifugation of heparinized whole blood, the cell pellet was re-suspended in NH_4_Cl-Tris solution for lysis of red blood cells, filtrated through nylon net and placed into 96-well plates. Leucocytes were repeatedly suspended in NH_4_Cl-Tris (H_2_O, NH_4_Cl, Tris-HCl, pH 7.5) and finally washed in FACS buffer (PBS, 0.5% BSA, 0.02% sodium azide, pH 7.45), then incubated for 20 min with Fc-block (BD Pharmingen, Heidelberg, Germany). Cells were then stained with fluorescence-conjugated monoclonal antibodies (BD Pharmingen, Caltag Laboratories GmbH, Hamburg, Germany), and propidium iodide (20 µg/ml) and washed with FACS buffer. Data were acquired using a FACS LSR II-HTS (Becton Dickinson, San Diego, USA). Live and dead cells were discriminated and the minimum number of living CD45+ cells (‘stopping gate’) was 3×10^4^. Data were compensated and analyzed using the FlowJo software (TreeStar, Inc. USA). Frequencies of granulocytes, monocytes, T-cells, B-cells and NK-cells were referred to the CD45+ cells, whereas frequencies of subtypes are given as proportion of the referring parent gate (e.g. CD4+/CD44+ is the proportion of CD44 positive cells within the CD4+ T cell cluster).

### Immunological Challenging Assay with *Listeria*

Infection of homozygous *Ifitm1* knockout and wildtype mice was performed with recombinant *Listeria monocytogenes* expressing ovalbumin (*L.m.*-Ova, [51]) or the parental wildtype *Listeria monocytogenes* (*L.m.*-wt). Brain Heart Infusion (BHI) medium was inoculated with *Listeria* stock solution and incubated at 37°C until an OD_600_ of 0.05–0.1. Subsequently, the *L.m.* suspension was diluted with PBS to the required concentration and infection of female mice (n  = 17 homozygous knockouts, n  = 9 wildtype littermates) was performed by intravenous (iv) injection into the lateral tail vein. Mice were infected with 50,000 *L.m.*-wt for the determination of bacterial burden or with 5,000 *L.m.*-Ova for analysis of T cell responses by intracellular cytokine staining (ICCS). Secondary infection was performed with a high dose of 250,000 *L.m.*-Ova 6 weeks after primary immunization with 5,000 *L.m.*-Ova.

For the determination of bacterial burden, mice were sacrificed on day 3 after primary infection or on day 2 after re-infection. Spleens and livers of infected mice were homogenized in the gentleMACS™ dissociator (Miltenyi Biotec, Germany) according to the supplier’s instructions. Dilutions of triton-lysed organ homogenates (from 1∶10 to 1∶1000) were plated on BHI agar plates, incubated at 37°C over night and the number of colony forming units (CFU) was determined.

Intracellular cytokine staining (ICCS) was performed on day 7 after primary infection and on day 5 after re-infection. Splenocytes were re-stimulated for 5 h with OVA_257–264_ (SIINFEKL) or LLO_190–201_ (NEKYAQAQPNVS). Intracellular cytokine stainings for IFNγ and TNFα were performed using the Cytofix/Cytoperm™ kit (BD Biosciences, USA) according to the supplier’s instructions. Data were acquired using the LSRII (BD Biosciences, USA) and analyzed using the FlowJo (Tree Star, USA) software.

### RNA Isolation and Microarray Based Gene Expression Profiling

Total RNA was isolated from lung tissue of *Ifitm1* deficient and control mice (n = 5 per group) at the age of 120–150 days employing the RNeasy Mini kit (Qiagen, Germany) including on-column digestion of remaining DNA. The RNA 6000 Pico Assay (Agilent) was used to assess RNA quality and samples with RIN values higher than 8 were used for microarray analysis. Total RNA (200 ng) was amplified using the GeneChip Whole Transcript (WT) Sense Target Labeling Assay (Affymetrix). Amplified cDNA (2.5 µg) was hybridized on Affymetrix Mouse Gene 1.0 ST arrays containing about 28 k gene-level probe sets. Staining and scanning was done according to the Affymetrix expression protocol. Expression console (Affymetrix) was used for quality control and to obtain annotated normalized RNA gene-level data (standard settings including sketch-quantile normalization). Data were filtered for low expressed genes (average expression <20 in both groups, wildtype and homozygous mutant animals) and for genes with fold-changes <1.1-fold. Statistical analysis and heat maps were generated in CARMAweb. Genewise testing for statistically significant differential expression was done employing the limma t-test and Benjamini-Hochberg multiple testing correction. Genes with p<0.01 (limma t-test) were further analyzed using GePS software (Genomatix). Array data is publically accessible from the Gene Expression Omnibus database under accession number GSE24744.

## Supporting Information

Figure S1 **ES cell screening PCR and Southern Blot analysis.** (A) Agarose gel electrophoresis of PCR products using ES-PCR-f1– GAC GTA AAC TCC TCT TCA GAC C and ES-PCR-r1– GGG AGA GAG TAG AGA AGT AAA GGC on genomic DNA from electroporated and G418 selected ES cells (indicated as ES-PCR in Fig. 1). The expected size of the PCR product (following homologous recombination) is 1.3 kb in length. The size marker in the first and last lane is a 1 kb ladder (MBI fermentas). A BAC clone containing the *lacZ* knockin *Ifitm1* allele was used as positive control (BAC control) for the screening PCR. The ES cell clone in lane 24 was used for blastocyst injection and the generation of chimeric mice (F0). (B) Southern blot hybridization of PvuII digested genomic tail DNA from littermate mice following excision of the neomycin selection cassette using *Cre* recombinase (see Fig. 1A). The blot was hybridized with probe sp as indicated in Fig. 1. It hybridizes to a genomic PvuII fragment of 4.2 kb in the wildtype allele and to a PvuII fragment of 2.2 kb in the targeted *Ifitm1^tm1IEG^* allele. A typical result is shown for a wildtype mouse (+/+), a heterozygous mutant (+/−) and a homozygous mutant mouse (−/−).
(TIF)

Figure S2 **Summary of sperm motility assays and measured parameters in fresh life sperm from Ifitm1^tm1IEG^ homozygous knockout animals (ko) and wildtype littermates (wt).** Top row: Velocity of average path (VAP in µm/s), velocity of straight line (VSL in µm/s), and velocity of the curvilinear path (VCL in µm/s). Middle row: Average lateral head displacement (ALH in µm), beat cross frequency (BCF in Hz), and straightness (STR  =  VSL/VAP). Bottom row: Linearity (LIN  =  VSL/VCL), percentage of motile sperm (motile.PCT in %), and percentage of progressive sperm (progressive.PCT in %). Blue boxes indicate the range between 25% and 75% percentile, the line inside the blue boxes indicates the median (50% percentile). The horizontal lines above and below the blue boxes indicate the lowest and highest value not classified as outlier. Outliers were defined as lying more than the 1.5fold the box length below the 25% percentile or above the 75% percentile and are indicated as dots and lines outside of the error bars. Statistically significant differences between homozygous knockout and wildtype mice in the sperm motility assay were not found.
(TIF)

Figure S3 **Immune response of** ***Ifitm1*** **homozygous mutant and wildtype mice following infection with** ***Listeria monocytogenes*** **.** (A) Shows the number of colony forming units (cfu) detected in liver and spleen of *Ifitm1^tm1IEG/tm1IEG^* (red bars) and *Ifitm1^wt/wt^* (blue bars) mice after primary infection with *Listeria monocytogenes*. We did not observe significant differences in the number of cfu between the organs of mutant and wildtype mice. (B) Shows the frequencies of Interferon gamma (IFNg, upper panel in B) and Tumor necrosis factor alpha (TNFa, lower panel in B) producing in CD4+ and CD8+ T cells after primary and secondary (recall) infection in Ifitm1*^tm1IEG/tm1IEG^* (red bars) and *Ifitm1^wt/wt^* (blue bars) animals. No significant differences in the concentrations of both cytokines were evident in the comparison between the two genotypes.
(TIF)

Figure S4 **Direct comparison of** ***lacZ*** **reporter gene expression via beta galactosidase staining (left panel) and of** ***Ifitm1*** **RNA in situ hybridization (right panel) in E9.5 embryos.** With both methods concordant expression was found in the somitic mesoderm, the neural tube, the developing gut up to the level of the anlage of the lung as well as in the ventral brain. However, gene expression detection using the beta galactosidase enzymatic staining is more sensitive than the RNA in situ hybridization method (compare, for example, the staining in the anterior somitic mesoderm and the ventral brain). Abbreviations: b – ventral brain region, la – anlage of the lung, PSM – presomitic mesoderm, s – somite.
(TIF)
