# Correction: *In Vivo* Functional Requirement of the Mouse *Ifitm1* Gene for Germ Cell Development, Interferon Mediated Immune Response and Somitogenesis

**DOI:** 10.1371/annotation/e53f8ba4-8e00-45ec-a051-e48d003f278a

**Published:** 2012-11-05

**Authors:** Ingeborg Klymiuk, Lukas Kenner, Thure Adler, Dirk H. Busch, Auke Boersma, Martin Irmler, Barbara Fridrich, Valérie Gailus-Durner, Helmut Fuchs, Nicole Leitner, Mathias Müller, Ralf Kühn, Michaela Schlederer, Irina Treise, Martin Hrabě de Angelis, Johannes Beckers

Barbara Fridrich was not included in the author byline. She should be listed as the seventh author and affiliated with Institute of Experimental Genetics and German Mouse Clinic, Helmholtz Zentrum München GmbH, Neuherberg, Germany. Her initials correctly appear in Author Contributions. 

