# Supplementary figures and images for: In Vivo Functional Requirement of the Mouse Ifitm1 Gene for Germ Cell Development, Interferon Mediated Immune Response and Somitogenesis
Source: PLoS One. 2012 Oct 24;7(10):e44609. doi: 10.1371/journal.pone.0044609 (PMC3480353; doi:10.1371/journal.pone.0044609)

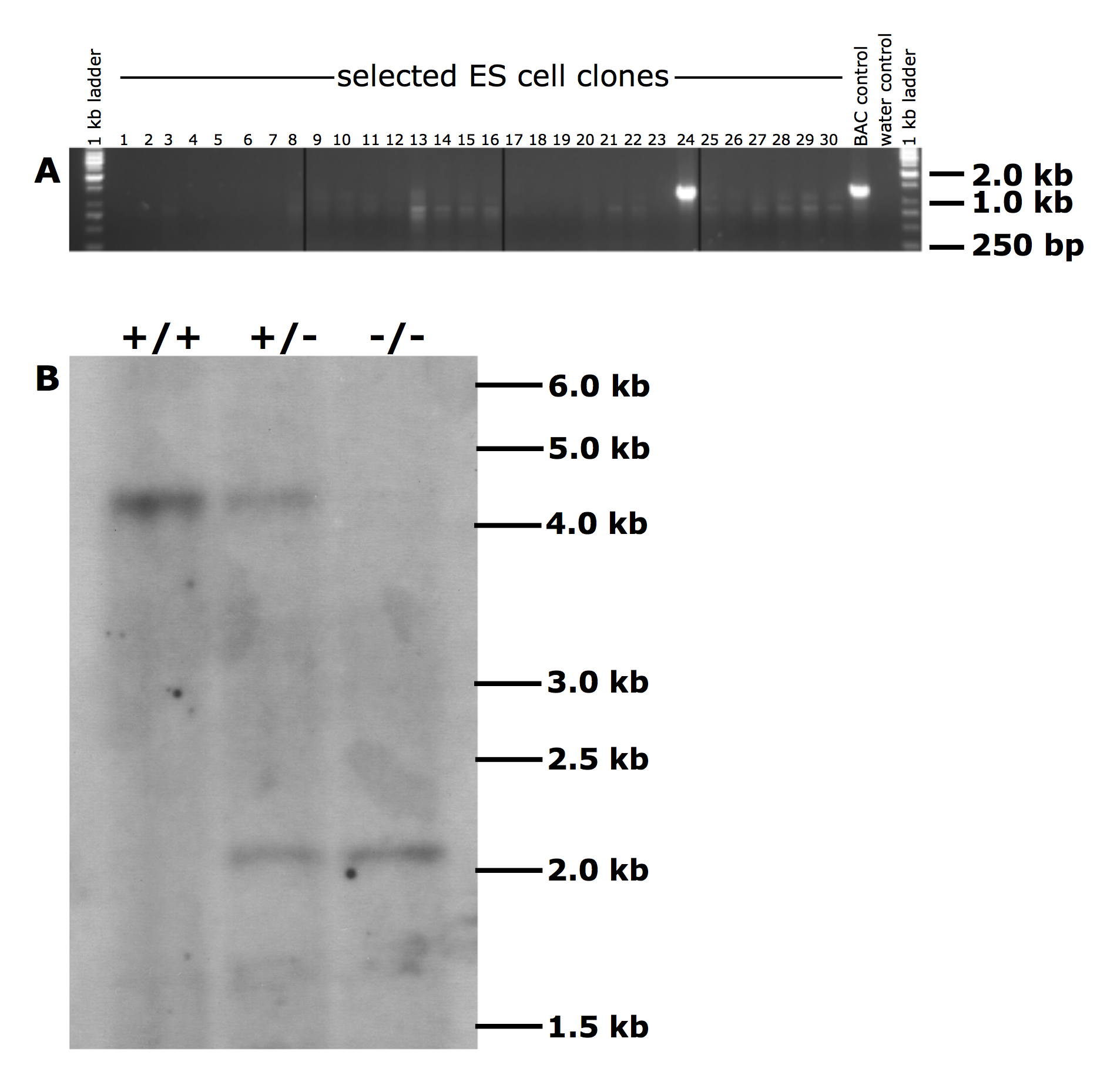

Supplement: Figure S1 — ES cell screening PCR and Southern Blot analysis. (A) Agarose gel electrophoresis of PCR products using ES-PCR-f1– GAC GTA AAC TCC TCT TCA GAC C and ES-PCR-r1– GGG AGA GAG TAG AGA AGT AAA GGC on genomic DNA from electroporated and G418 selected ES cells (indicated as ES-PCR in Fig. 1). The expected size of the PCR product (following homologous recombination) is 1.3 kb in length. The size marker in the first and last lane is a 1 kb ladder (MBI fermentas). A BAC clone containing the lacZ knockin Ifitm1 allele was used as positive control (BAC control) for the screening PCR. The ES cell clone in lane 24 was used for blastocyst injection and the generation of chimeric mice (F0). (B) Southern blot hybridization of PvuII digested genomic tail DNA from littermate mice following excision of the neomycin selection cassette using Cre recombinase (see Fig. 1A). The blot was hybridized with probe sp as indicated in Fig. 1. It hybridizes to a genomic PvuII fragment of 4.2 kb in the wildtype allele and to a PvuII fragment of 2.2 kb in the targeted Ifitm1tm1IEG allele. A typical result is shown for a wildtype mouse (+/+), a heterozygous mutant (+/−) and a homozygous mutant mouse (−/−). (TIF) [file pone.0044609.s001.tif]

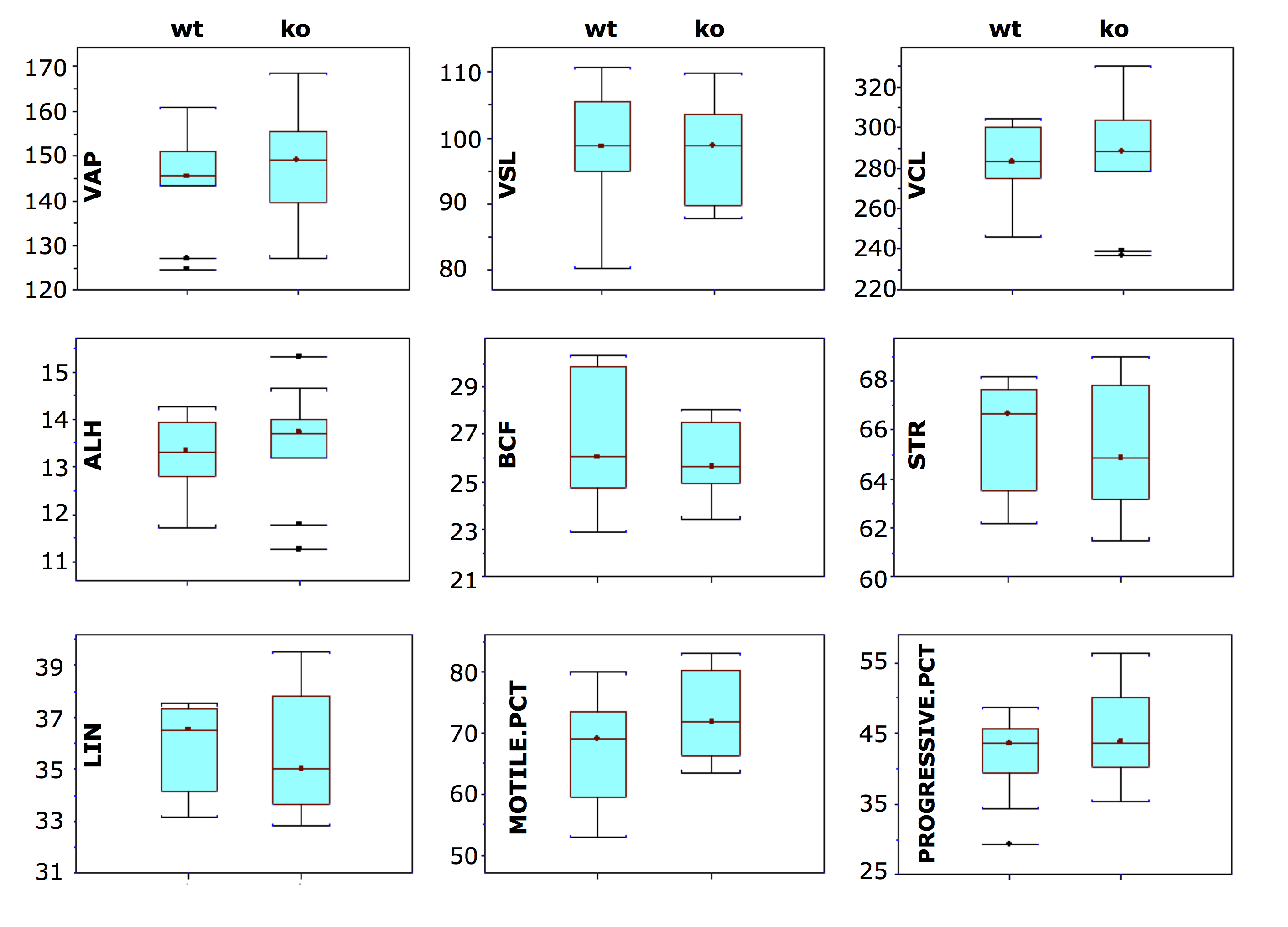

Supplement: Figure S2 — Summary of sperm motility assays and measured parameters in fresh life sperm from Ifitm1tm1IEG homozygous knockout animals (ko) and wildtype littermates (wt). Top row: Velocity of average path (VAP in µm/s), velocity of straight line (VSL in µm/s), and velocity of the curvilinear path (VCL in µm/s). Middle row: Average lateral head displacement (ALH in µm), beat cross frequency (BCF in Hz), and straightness (STR = VSL/VAP). Bottom row: Linearity (LIN = VSL/VCL), percentage of motile sperm (motile.PCT in %), and percentage of progressive sperm (progressive.PCT in %). Blue boxes indicate the range between 25% and 75% percentile, the line inside the blue boxes indicates the median (50% percentile). The horizontal lines above and below the blue boxes indicate the lowest and highest value not classified as outlier. Outliers were defined as lying more than the 1.5fold the box length below the 25% percentile or above the 75% percentile and are indicated as dots and lines outside of the error bars. Statistically significant differences between homozygous knockout and wildtype mice in the sperm motility assay were not found. (TIF) [file pone.0044609.s002.tif]

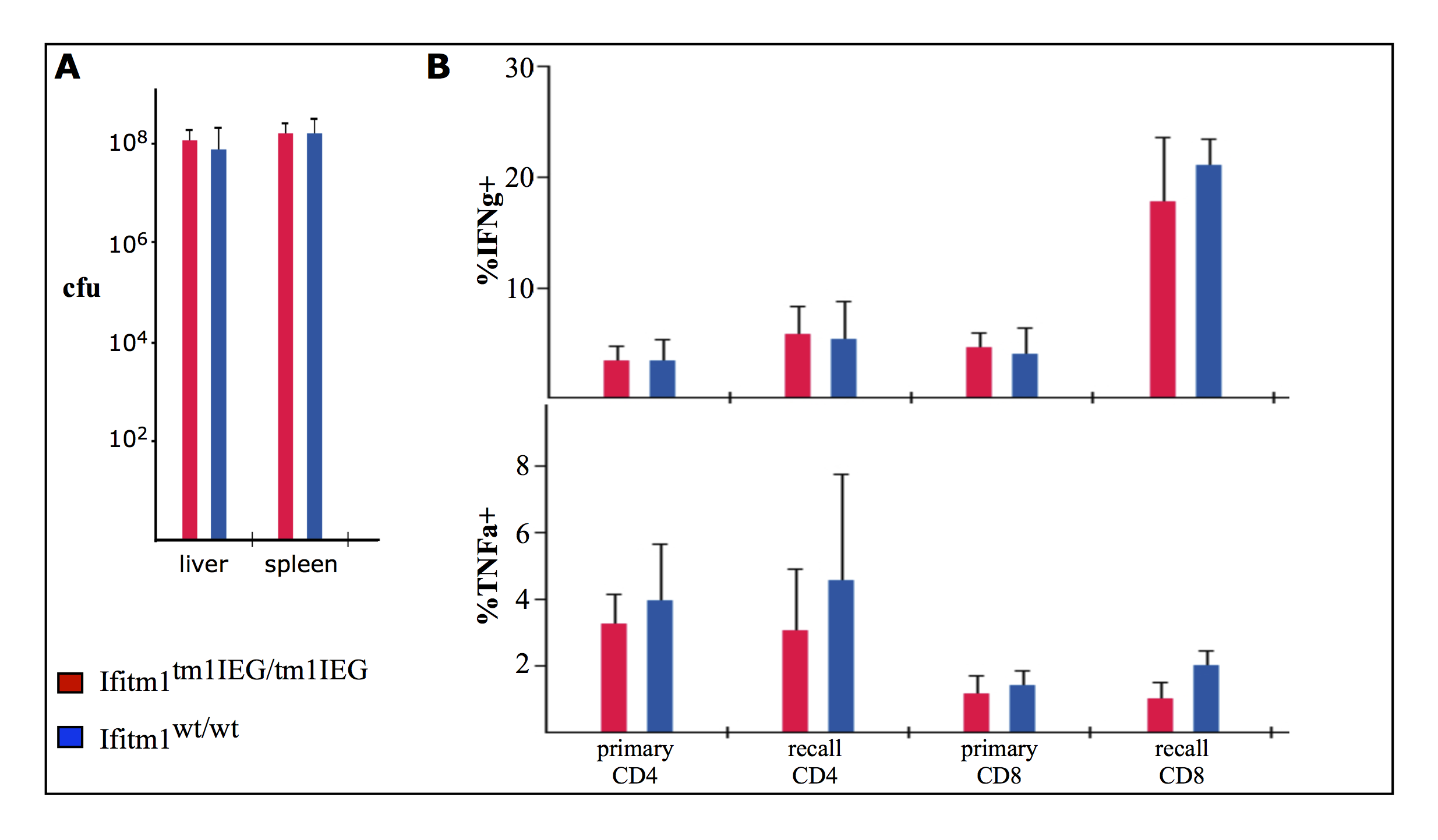

Supplement: Figure S3 — Immune response of Ifitm1 homozygous mutant and wildtype mice following infection with Listeria monocytogenes . (A) Shows the number of colony forming units (cfu) detected in liver and spleen of Ifitm1tm1IEG/tm1IEG (red bars) and Ifitm1wt/wt (blue bars) mice after primary infection with Listeria monocytogenes. We did not observe significant differences in the number of cfu between the organs of mutant and wildtype mice. (B) Shows the frequencies of Interferon gamma (IFNg, upper panel in B) and Tumor necrosis factor alpha (TNFa, lower panel in B) producing in CD4+ and CD8+ T cells after primary and secondary (recall) infection in Ifitm1tm1IEG/tm1IEG (red bars) and Ifitm1wt/wt (blue bars) animals. No significant differences in the concentrations of both cytokines were evident in the comparison between the two genotypes. (TIF) [file pone.0044609.s003.tif]

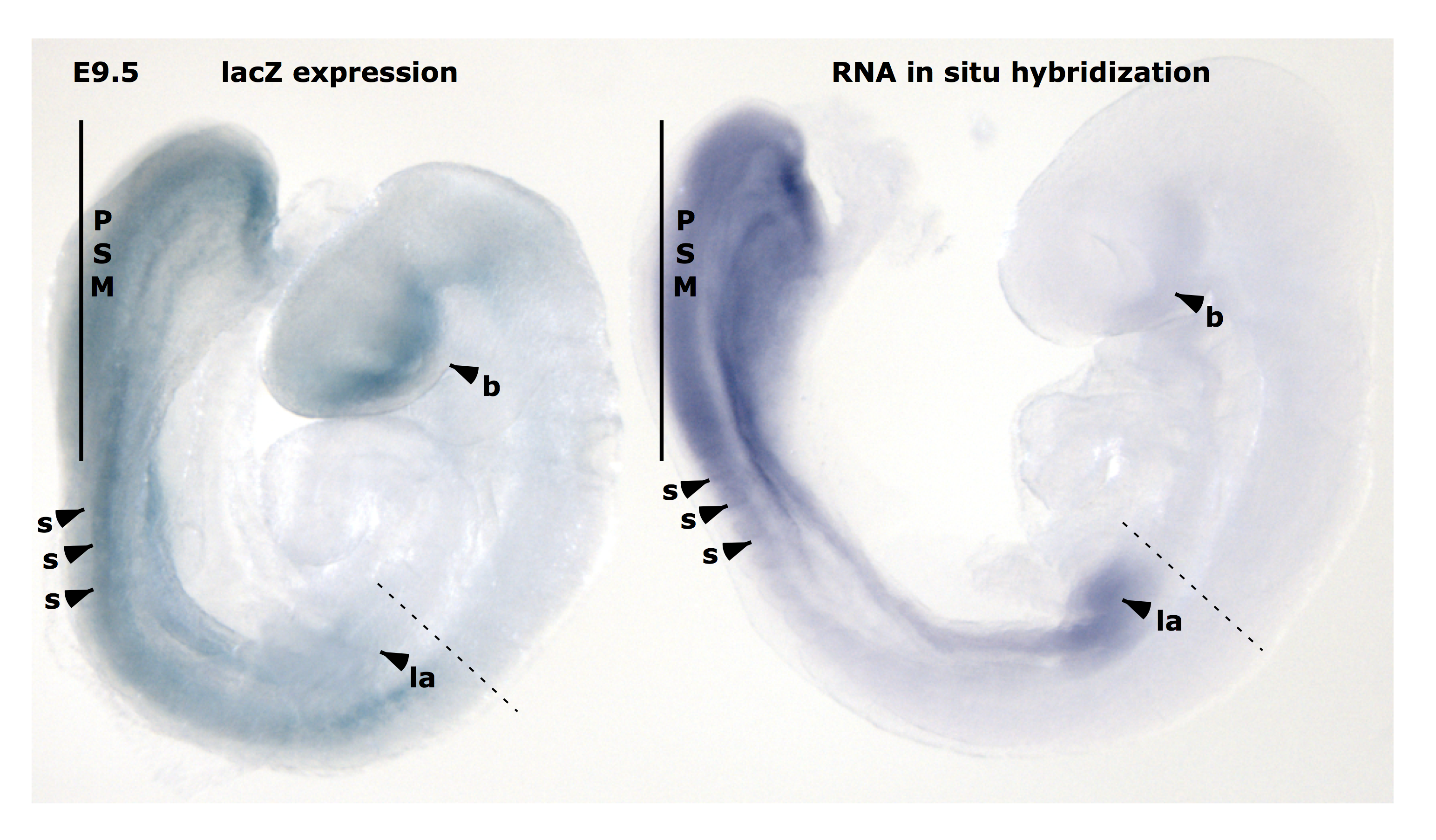

Supplement: Figure S4 — Direct comparison of lacZ reporter gene expression via beta galactosidase staining (left panel) and of Ifitm1 RNA in situ hybridization (right panel) in E9.5 embryos. With both methods concordant expression was found in the somitic mesoderm, the neural tube, the developing gut up to the level of the anlage of the lung as well as in the ventral brain. However, gene expression detection using the beta galactosidase enzymatic staining is more sensitive than the RNA in situ hybridization method (compare, for example, the staining in the anterior somitic mesoderm and the ventral brain). Abbreviations: b – ventral brain region, la – anlage of the lung, PSM – presomitic mesoderm, s – somite. (TIF) [file pone.0044609.s004.tif]
